# The Risk-Taking Propensity of Construction Workers—An Application of Quasi-Expert Interview

**DOI:** 10.3390/ijerph15102250

**Published:** 2018-10-15

**Authors:** Banus Kam Leung Low, Siu Shing Man, Alan Hoi Shou Chan

**Affiliations:** Department of Systems Engineering and Engineering Management, City University of Hong Kong, Hong Kong, China; ssman6-c@my.cityu.edu.hk (S.S.M.); alan.chan@cityu.edu.hk (A.H.S.C.)

**Keywords:** construction safety, individual factor, organisational factor, quasi-expert interview, risk-taking propensity

## Abstract

High accident rates have been a complicated and persistent problem in the Hong Kong construction industry. This situation has stimulated this investigation into factors that influence the risk-taking propensity of construction workers. However, interviewing workers who had a bad experience is problematic because changes in attitude and perception may occur as a result of such an experience. Using quasi-expert interviews can reduce this problem. The objective of this study was to identify factors that influence the risk-taking propensity of construction workers. Semi-structured interviews were conducted with 16 safety professionals all with accident inspection experience and six super-safe workers with no incident record for the past five years. Seven factors that affect the risk-taking propensity of construction workers were successfully identified. Each factor is thoughtfully discussed, and this study shows that quasi-expert interview is a pragmatic approach for deepening the understanding of risk-taking propensity among construction workers. Findings of this study will hopefully help and encourage further quantitative research on the risk-taking propensity of construction workers with different perspectives.

## 1. Introduction

Despite the important contributions of the construction industry to developing and developed countries in terms of gross domestic product (GDP) [1] and employment opportunities, it has long been considered one of the most dangerous industries. High accident rates in the construction industry around the world are of great concern, particularly that in Hong Kong. Hong Kong’s construction industry has a fatality accident rate per thousand workers which is 2.23 times that of Japan [2] and 2.43 times that of Singapore in 2017 [3]. Human factors play an important role in accident occurrence and are closely associated with behaviour-based safety (BBS) among building workers [4,5]. Understanding the risk-taking propensity of workers is critical because it provides clues about the relationship between behavioural outcomes and accident proneness [6,7].

Risk taking is common, yet the risk-taking propensity among construction workers is distinct. Many factors can affect the risk-taking propensity of workers, and they can generally be categorised as organisational [8] and individual factors [9]. Although many previous studies were conducted to test factors in the model of unsafe behaviour for construction workers, their findings heavily relied on statistical analyses. For instance, Fogarty and Shaw [10] investigated the prediction of unsafe behaviour with safety climate as a predictor. Jiang, et al. [11] employed system dynamics modelling to understand the causation of unsafety behaviours of construction workers. These studies are useful in examining statistical relationships between variables. However, they cannot explain why construction workers possess risk-taking propensity.

Certain attitudes are believed to be greatly affected by and vary according to circumstances. For instance, attitude towards risk is likely to change immediately after an incident has occurred due to the altered mental state of workers after accidents or negative experiences [12]. This change can occur even when workers witness a workplace accident [13] and can result in negative effects on survey results by not reflecting the true attitude of workers towards risk. Another factor that can affect study results is de-biasing effect which is the suppression of unrealistic optimism by having a negative experience [14]. Such changes in bias with regard to unrealistic optimism were reported for the effect of frequency of accidents on the attitude of children towards risk taking [15]. This adverse effect of negative experience on study results was recognised. 

In this study, the risk-taking propensity of construction workers refers to their tendency to engage in benefit-seeking actions at work despite potential negative results. This study aimed to understand factors that influence their risk-taking propensity at work by conducting quasi-expert interviews. The findings of this study are expected to provide a theoretical insight into risk-taking propensity and offer practical recommendations to reduce the risk-taking propensity of construction workers.

## 2. Literature Review

How construction accidents happened and what type of these accidents are have long been investigated with different reporting systems and techniques [16]. For instance, Tixier, et al. [17] proposed a natural language processing system for extracting precursors and outcomes from unstructured injury reports. To understand why construction accidents occurred is important for the reduction of construction accidents. There are two domains of theories attracting attention of researchers, namely and theories of human error and the theories of accident causation. Heinrich Domino theory of accident causation is one of the well-known theories of accident causation and it is based on five sequential dominos; (1) ancestry and social environment; (2) fault of person (carelessness); (3) unsafe act and/or mechanical or physical condition; (4) accident; (5) injury [18]. The Heinrich Domino theory states that if the first domino (ancestry and social environment) falls, the following dominos will fall in sequence. This theory implies that the avoidance of construction accidents can be achieved if the chain of sequence is disturbed. For example, the unsafe act of construction workers can be eliminated to prevent the accidents and associated injuries. However, the Heinrich Domino theory was blamed for its simplifying the human behaviour control in accidents, leading to more emphasis to be put on the management role in accident prevention [19].

Different from Heinrich Domino theory, the theories of human error do not attribute accidents to unsafe human behaviour, but to the design of workplace tasks that do not consider the limitations of human [20]. Petersen [21] proposed a multiple causation model that focuses on management system rather than individuals. In the multiple causation model, unsafe acts and unsafe conditions were attributable to different sub-causes. By eliminating these sub-causes, unsafe act and unsafe condition can be prevented. The needs to improve training and inspection procedures, and to make better assignment of responsibilities, and pre-task planning by supervisors was stressed [21].

Fleming and Lardner [22] found that approximately 80% of accidents are caused by unsafe human behaviour. For improving construction safety, researchers have focused on understanding unsafe behaviour of construction workers. Choudhry and Fang [23] examined the reasons for construction worker unsafe behaviour and found that workers were involved in unsafe behaviour because of: to exhibit of being ‘tough guys’, a lack of safety awareness; co-workers’ attitudes; work pressure; and other organizational, economic and psychological factors. Also, Fang, et al. [24] proposed a cognitive model that adopted a five-stage form for explaining constriction worker unsafe behaviour. The five stages included obtaining information, understanding information, perceiving responses, selecting a response, and taking action, with obtaining information and selecting a response as the two key stages. Khosravi [25] conducted a quantitative study to test a new model for understanding the factors influencing unsafe behaviour in construction industry and found that physical condition had the highest correlation with the overall safety performance. However, there is a lack of studies on the risk-taking propensity of construction workers in the relevant literature. This study aimed to understand the factors that influence their risk-taking propensity at work by conducting quasi-expert interviews. 

## 3. Methodology 

This study employed a qualitative approach to obtaining the thoughts of participants about the risk-taking propensity of construction workers. The details of methodology design are discussed below. 

### 3.1. Interview and Question Design

Face-to-face quasi-expert interviews were conducted because the relatively objective notions and knowledge of experts were considered to avoid potential bias of the victims of accident cases [26,27]. All the interviewees voluntarily participated in the interviews in the presence of the research staff only. They were assured the whole study was conducted by university staff with high level of data anonymity, security and confidentiality. Two interview groups were used to obtain comprehensive qualitative data about risk-taking propensity in this study, namely, accident and super-safe groups. In the accident group, the accident cases which were construction accident reports were involved. Safety frontline officers and related managerial safety professionals, who were experienced in handling accident inspections in Hong Kong construction projects, possessed a certain understanding of these accidents and knew the victims as colleagues before the occurrence of the accidents and as clients after a detailed investigation of the accidents, were interviewed to obtain their relatively objective comments on construction accidents. In the super-safe group, super-safe workers had no any official accident records in the last five years and were front-line workers who could provide their experience and opinions about not taking risks at work. Accident cases and super-safe workers were randomly selected from 10 construction companies that are contracted for different types of construction projects in Hong Kong that have certain representativeness in the industry. Specifically, a number was assigned to all potential accident cases and super-safe workers and then a computer-based program was used to generate a random number table to select accident cases and super-safe workers for the sample. There were only two interviewers, the first author and a research staff member, who had more than 10 years of working experience in the construction field to ensure that they fully understand technical terms and/or specific procedures referred to by the participants and the circumstance of the accident or situation confronting the unfortunate person or persons involved in the accident.

A semi-structured interview guide was developed for interviewers wherein they ask predetermined open questions to obtain comprehensive responses from participants. Questions used for both the accident and super-safe groups were categorised in a three-layer sequence as follows: Opening questions, follow-up questions and in-depth discussions. Opening questions introduced the participants to the general purpose of the study, and follow-up questions covered contents of the survey. In-depth discussions concentrated on uncovering and probing the underlying reasons for risk-taking propensity. 

In the accident group, the opening question was ‘Could you briefly describe the context of an accident case you have encountered?’ This question provided basic information about the reported accident case and general area of risk taking. Follow-up questions and in-depth discussions, however, continued with an example to further investigate the underlying causes of the accident and provide information and insight for the interviewer. 

In the super-safe group, the opening question was designed to praise their performance over the past five years, for example, ‘You have zero reported accidents over the past five years. How did you do that?’ and ‘May I call you a super-safe worker?’ These techniques created an atmosphere that helped the interviewees express themselves freely. As the two groups of participants were from different backgrounds, follow-up questions used for the accident group were slightly different from those used for the super-safe group. The super-safe group questions concentrated on the reasons for not taking risks at work. In spite of this difference, the context of all the questions was related to the factors that influence the risk-taking propensity of construction workers under examination. Table 1 shows the details of interview questions for the accident group and the super-safe group.

### 3.2. Participants

Thirty-one accident cases were used in the interviews with 16 safety professionals (accident group) and six construction workers without any incident records in the last five years (super-safe group). Table 2 shows the general demographic information of accident cases and super-safe workers in this study. In the accident group, the majority of victims were male (96.8%), aged above 30 years (93.6%), held an education level of primary school (62.1%) and had more than 1 year of working experience in the construction industry (96.8%). In the super-safe group, the number of male and female participants was equal. Most of them were aged over 51 years (83.3%), and all participants were married.

### 3.3. Investigating Factors

Voice recording was used during interviews. Qualitative data were obtained from transcriptions of the recordings to provide quotes, which help in understanding the factors that affect risk-taking propensity. Constant comparative approach was specifically adopted for data analysis to generate in-depth meanings [28]. For example, a response from the accident group *‘… they noticed no safety officer doing safety inspections during that period’* was coded as a ‘Safety Supervision and Inspection’ theme because a lack of safety supervision and inspection may result in taking risks. A response from another participant ‘*There was not enough site supervision. There were some problems on the site, but nobody cared about them …*’ was examined and compared to determine whether it was similar to the previously identified themes. If so, the response was coded as a ‘Safety Supervision and Inspection’ theme. Otherwise, the response was coded as a new theme. Related pieces of conversation were identified and stated.

## 4. Research Findings and Discussions

Qualitative data were analysed systematically through constant comparative approach to extract in-depth understanding of each participant’s viewpoint on risk-taking propensity. Risk-taking propensity was found to be a combination of various contributing factors. Generally, two domains of factors that affect risk-taking propensity of construction worker were organisational and individual factors. Organisational factors involved safety supervision and inspection, safety culture, social influence and workplace condition. Individual factors included attitude towards risk, risk perception and perceived behavioural control. These factors are discussed in depth below. A list of sample responses from the accident group and the super-safe group is shown in Table 3. Also, the results of coding for accident group and super-safe group are shown in Table 4.

### 4.1. Safety Supervision and Inspection

In this study, safety supervision and inspection refer to frequency, broadness and depth of safety supervisions and inspections on site. Improper safety supervision and inspection, like ‘No close safety supervision’, might lead to more risk-taking behaviours such that they may subsequently result in construction accidents. The following statement from the accident group was reported: *‘… they noticed no safety officer doing safety inspections during that period’.* This finding is consistent with that of Fung, et al. [29] who found that safety supervision is highly negatively correlated with risk taking behaviour among Hong Kong construction workers. Safety professionals agreed that *‘… sometimes, we face a shortage of personnel to supervise safety in the workplace …’*, indicating a manpower-shortage problem in the Hong Kong construction industry. One super-safe worker reported that ‘*We were discouraged from engaging in unsafe work practices when our supervisors were inspecting. Therefore, a regular inspection by our supervisors is very important. It may not be a proactive way to improve our safety performance, but at least it worked’.* Such a response indicates that super-safe workers behave passively during an inspection. A previous study of Hoła, et al. [30] proposed a methodology of classifying the causes of occupational accidents involving construction scaffolding using Pareto-Lorenz analysis and found that in the group of organizational causes, first and foremost, is a lack of direct supervision by a construction manager or executive manager during the performance of work. Therefore, the construction industry and concerned authorities should be aware of the problems related to safety supervision and inspection. They should also employ additional human resources to ensure adequate safety supervision and inspection for construction workers, thereby reducing their risk-taking propensity. 

### 4.2. Safety Culture

Safety culture constitutes values and beliefs that involve interaction between organisations and individuals [31]. The present study found that safety culture might affect risk-taking propensity of construction workers. One safety professional echoed the responses of the construction workers as follows: *‘The safety culture of the team was poor …. For instance, no person in the team was assigned to clean up debris and or pick up cables that were left lying on the floor for few days. Many workers replied that cleaning debris was not their responsibility …’* By contrast, a super-safe worker stated the following about the safety culture of his working team: ‘*… I feel my team was concerned about being considerate and responsible for coworkers …’* This statement reflects the important effects that safety culture can exert on the attitudes and actions of Hong Kong construction workers. For example, a negative safety culture can influence a worker to take risks through social pressure. This social pressure is likely to be heightened in closely bonded work communities. To provide a positive safety culture for construction workers, construction safety weeks are advised to be organised for them, and activities may include safety carnivals, conferences and safety award presentations.

### 4.3. Social Influence

In this study, social influence refers to subjective norms of the participants in the safety aspect. It was also found as a factor that affects risk-taking propensity of construction workers, similar to the findings of Zohar and Luria [32] that social influence can influence the safety performance of individuals. The accident group demonstrated much tendency to take risks. The following statements were from a safety officer in the accident group ‘*… they just followed the practice (unsafe practice) of the group …*’ and *‘they think they were not the only one who did that (unsafe behaviour) … there were other people (workers) who did the same …’* These quotes are victim responses during accident investigations and are typical examples of how workers complied with unsafe norms prevailing in the workplace. Construction projects in Hong Kong are generally extensive, called ‘mega’, and involve a large work force for each project. Intensive work under time and cost pressure is likely to result in negative social norms regarding unsafe practices within an organisation [33]. The present study revealed the influence of social influence and norms on the risk-taking propensity of construction workers, but not much research has been done on the formation of such unacceptable norms in Hong Kong’s construction industry. 

### 4.4. Workplace Condition

In this study, workplace condition is defined as the housekeeping of a construction site. Complaints about insufficient lighting, limited space and debris problems were reported to safety officers in the accident group. Statements like *‘the site area was pretty dark and I usually couldn’t see the floor clearly …’* indicate that insufficient lighting may cause workers to work in a risky environment. Moreover, many workers argued that cleaning debris was not their responsibility. They continued to work under unclean conditions, which may cause accidents. Other workers identify limited space in their workplace as another driver of risk-taking behaviour. One respondent reported that ‘*The workplace was located 30 m above ground, the working platform was small and its loading capacity was just enough to support the drilling rig’*. These findings are consistent with that of Ghosh, et al. [34] who found that poor workplace conditions are related to the risk-taking tendency and occupational injuries of workers. In addition, these findings imply that providing a clean and safe workplace for construction workers can reduce their risk-taking propensity.

### 4.5. Attitude towards Risk

Attitude towards risk refers to a person’s positive or negative evaluation of risks at work. In the accident group, safety professionals mentioned that ‘*… they knew about the consequences of engaging in risky behaviour. These workers think risks are accompanied with certain benefits, thereby taking risks at work’*. Such response clearly indicates a positive risk attitude of workers who have accidents. In the super-safe group, respondents reported that ‘*… I think risks at work are harmful to our safety and health so I did not take risks at work …’* This response shows super-safe workers hold a negative risk attitude. These findings imply that attitude towards risk is a factor of the risk-taking propensity of construction workers. Previously, Wang and Yuan [35] identified factors affecting risk attitudes of construction project contractors. However, no studies have been conducted to identify factors that influence attitude towards risk among construction workers. Future research may focus on this issue.

### 4.6. Risk Perception

Risk perception refers to subjective judgement about a risk. In the accident group, one interviewee reported that ‘*… workers did not follow safety procedure because they did not think doing so is dangerous …’* This statement implies that low risk perception may lead to risk-taking propensity of construction workers. By contrast, in the super-safe group, a worker reported that ‘*… I did not take risks, such as not using safety helmets because I believe not using safety helmet can lead to serious injuries …*’ This claim indicates workers who have a high level of risk perception tend to avoid risks at work. These findings are in agreement with that of Arezes and Miguel [36] who found that worker risk perception is a significant predictor of using hearing protection devices. Risk perception has received increasing attention from safety searchers. For instance, Bohm and Harris [37] explored risk perception of dumpers and its relationship to their risk-taking behaviour using a paired comparison technique. They found that risk perception was negatively related with risk-taking behaviour. The present study advocated that risk perception is one factor influencing the risk-taking propensity of construction workers. Safety training should be given to construction workers to increase their risk perception so that their risk-taking propensity can be reduced.

### 4.7. Perceived Behavioural Control

Perceived behavioural control refers to the extent to which workers perceive the ease or difficulty of taking risks at work. In this study, perceived behavioural control affects risk-taking propensity. In the accident group, one respondent reported that ‘*… workers did not use a safety harness to work at height because they think it is easy to complete the task without safety measures …’* For the super-safe group, a response ‘*… I think it is difficult to take risks at work …’ was obtained.* These statements indicate that workers with a high level of perceived behavioural control tend to take risks at work. According to the theory of planned behaviour [38], the intention of performing a behaviour is determined by perceived behavioural control over that behaviour. This study supported this theory in the context of construction safety. 

## 5. Conclusions

In this qualitative study, seven factors associated with construction worker risk-taking propensity were identified using quasi-expert interviews. Specifically, organization factors (including safety supervision and inspection, safety culture, social influence, and workplace condition) and individual factors (including attitude towards risk, risk perception, perceived behavioural control) were found to influence construction worker risk-taking propensity. The findings of this study help explain the risk-taking propensity of construction workers. The identification of factors affecting risk-propensity of construction workers may help industry stakeholders to allocate resources properly for improving safety performance of construction workers.

## Figures and Tables

**Table 1:** Interview questions for the accident group and the super-safe group.

	Accident Group	Super-Safe Group
Opening questions	*Could you briefly describe the context of an accident case you have encountered?*	*You have zero reported accidents over the past five years. How did you do that?*
		*May I call you a super-safe worker?*
In-depth questions	*Could you explicitly describe the reasons for the worker to take risks at work during the incident period?*	*Could you explicitly describe the reasons for not taking risks at work?*
	*Could you further describe the safety supervision and inspection during the incident period?*	*Could you further describe the safety supervision and inspection during your servicing period?*
	*Could you further describe the safety culture during the incident period?*	*Could you further describe the safety culture during your servicing period?*
	*Could you further describe the social influence of the worker during the incident period? What were the social norms regarding safety that s/he espoused?*	*Could you further describe the social influence of you during your servicing period? What were the social norms regarding safety that you espoused?*
	*Could you further describe the workplace conditions during the incident period?*	*Could you further describe the workplace conditions during your servicing period?*
	*Could you further describe the attitude of the worker towards risk during the incident period? Did s/he have any risky ideals at work?*	*Could you further describe the attitude of you towards risk during your servicing period? Did you have any risky ideals at work?*
	*Could you further describe the risk perception of the worker during the incident period?*	*Could you further describe the risk perception of you during your servicing period?*
	*Could you further describe the perceived behavioural control of the worker during the incident period? Was s/he full of confidence or not?*	*Could you further describe the perceived behavioural control of you during your servicing period? Were you full of confidence or not?*
Ending Question	*Do you have anything to add?*	*Do you have anything to add?*

**Table 2:** General demographic information of accident cases and super-safe workers.

Demographic Information	Accident Group (*n* = 31)	Super-Safe Group (*n* = 6)
Gender		
Male	96.8%	50.0%
Female	3.2%	50.0%
Age		
18–30 years old	6.5%	
31–40 years old	19.4%	
41–50 years old	32.3%	16.7%
Over 51 years old	41.9%	83.3%
Education Level		
Primary school or below	62.1%	66.7%
Middle school or above	37.9%	33.3%
Marital Status		
Single	13.8%	
Married	79.3%	100.0%
Divorced or separated	6.9%	
Work Experience		
1 year or less	3.2%	
1 to less than 3 years	6.5%	
3 to not more than 10 years	16.1%	16.7%
10 to less than 20 years	54.8%	83.3%
20 years or more	19.4%	
Number of Dependents		
None	6.9%	
1 to 2	41.4%	66.7%
3 to 4	51.7%	16.7%
More than 4		16.7%
Employment Type		
Employee of main contractor	3.2%	16.7%
Employee of subcontractor (S/C)	67.7%	83.3%
Employee of third tier subcontractors	6.5%	
Broker-type	22.6%	

**Table 3:** A list of sample responses from the accident group and the super-safe group.

Factor	Group	Example Quotes
Safety Supervision and Inspection	Accident Group	*‘… they noticed no safety officer doing safety inspections during that period’.*
		*‘… there was not enough site supervision. There were some problems on the site but nobody cared about them …’*
		*‘… they noticed that the foreman or the consultant’s representative was not present …’*
		*‘… sometimes, we face a shortage of personnel to supervise safety in the workplace …’*
		*‘… the site actually was operated without the supervisor’s involvement’.*
	Super-safe Group	*‘The safety officers patrolled the work site frequently’.*
		*‘We were discouraged from engaging in unsafe work practices when our supervisors were inspecting. Therefore, a regular inspection by our supervisors is very important. It may not be a proactive way to improve our safety performance, but at least it worked’.*
		*‘I know that the equipment has been well inspected by the relevant safety officers …’*
Safety Culture	Accident Group	*‘The safety culture of the team was poor … For instance, no person in the team was assigned to clean up debris and or pick up cables that were left lying on the floor for few days. Many workers replied that cleaning debris was not their responsibility …’*
		*‘… the team had a poor safety culture …’*
		*‘… there was no empathy in the team and this situation is getting worse’.*
		*‘they didn’t want to be isolated or blamed by others for asking for extra safety measures’.*
	Super-safe Group	*‘… I feel my team was concerned about being considerate and responsible for coworkers …’*
		*‘My group had a team spirit in safety … I feel more comfortable to work safely if the group has a team spirit …’*
		*‘My groupmates understood my safe work practices and did not blame me for working slow …’*
Social Influence	Accident Group	*‘they think they were not the only one who did that (unsafe behaviour) … there were other people (workers) who did the same …’*
		*‘… they just followed the practice (unsafe practice) of the group …’*
	Super-safe Group	*‘I followed safety practices because co-workers did so too …’*
		*‘If I work unsafely, other workers blame me …’*
		*‘I did not want to get others angry because of my unsafe behaviour …’*
Workplace Condition	Accident Group	*‘the site area was pretty dark and I usually couldn’t see the floor clearly …’*
		*‘… the workplace space was insufficient for us to work properly …’*
		*‘The workplace was located 30 m above ground, the working platform was small and its loading capacity was just enough to support the drilling rig’*
	Super-safe Group	*‘The workplace was tidy and had sufficient lighting …’*
		*‘I can see the access points to many locations in the site …’*
Attitude towards Risk	Accident Group	*‘… they knew about the consequences of engaging in risky behaviour. These workers think risks are accompanied with certain benefits, thereby taking risks at work …’*
		*‘… they think taking risks is not a bad idea’*
		*‘… they like taking risks at work …’*
	Super-safe Group	*‘… I think risks at work are harmful to our safety and health so I did not take risks at work …’*
		*‘… I think working unsafely is unwise …’*
Risk Perception	Accident Group	*‘… workers did not follow safety procedure because they did not think doing so is dangerous …’*
		*‘… they think they did it before, so doing it again would not be risky … However, an accident did happen …’*
		*‘… he did not perceive any risks in what he is about to do …’*
	Super-safe Group	*‘… I did not take risks, such as not using safety helmets because I believe not using safety helmet can lead to serious injuries …*’
		*‘… I think taking risks is very likely to result in accidents …*’
		*‘… I worry about the accidents which are caused by taking risks …*’
Perceived Behavioural Control	Accident Group	*‘… workers did not use a safety harness to work at height because they think it is easy to complete the task without safety measures …’*
		*‘… they always felt confident in taking risks …’*
	Super-safe Group	*‘… I think it is difficult to take risks at work …’*
		*‘… I have no ability to take risks …’*

**Table 4:** Results of coding for the accident group and the super-safe group.

Groups	Categories	Subcategories	Codes	Frequency
Super-safe Group	Organizational Factors	Safety Supervision and Inspection	Infrequent Safety Inspection	35
			No Close Safety Supervision	30
		Safety Culture	Bad Safety Culture	32
			Blaming Culture about Using Safety Measures	26
		Social Influence	Subjective Norms toward Unsafe Practice	31
			Compliance to Unsafe Practice of Co-workers	24
		Workplace Condition	Poor House Keeping	21
			Limited Workspace	18
			Insufficient Lighting	15
	Individual Factors	Attitude towards Risk	Preference for Risks	35
		Risk Perception	Low Risk	23
			No Danger	10
		Perceived Behavioural Control	Feeling of Ease	25
Super-safe Group	Organizational Factors	Safety Supervision and Inspection	Frequent Safety Inspection	13
			Close Safety Supervision	11
		Safety Culture	Good Safety Culture	11
			Encouragement to Use Safety Measures	9
		Social Influence	Subjective Norms toward Safe Practice	8
			Compliance to Safe Practice of Co-workers	8
		Workplace Condition	Good House Keeping	6
			Sufficient Work Space	4
			Sufficient Lighting	3
	Individual Factors	Attitude towards Risk	No Preference for Risks	11
		Risk Perception	High Risk	6
			Danger	4
		Perceived Behavioural Control	Feeling of Difficulty	6
